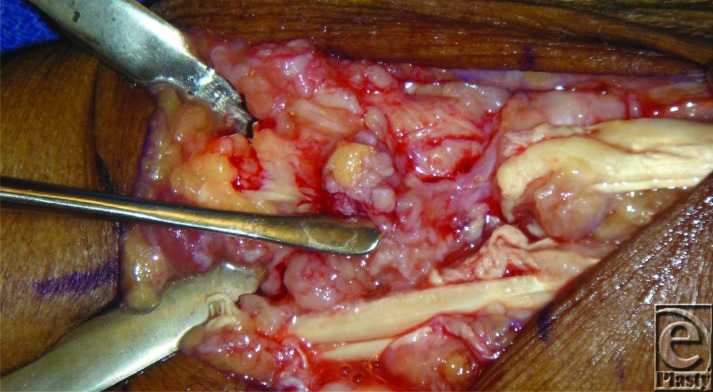# Rheumatoid Arthritis

**Published:** 2013-02-11

**Authors:** Keith E. Follmar, Justin M. Broyles, Jaimie T. Shores

**Affiliations:** Department of Plastic and Reconstructive Surgery, The Johns Hopkins University, Baltimore, Md

**Figure:**
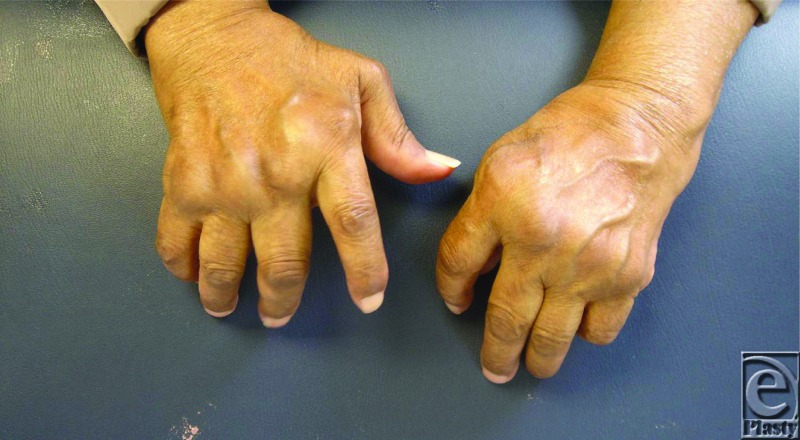


## DESCRIPTION

A 62-year-old right-hand-dominant retired woman presents 1 week after sudden loss of ability to actively extend her left small and ring fingers. The patent has a 4-year history of rheumatoid arthritis (RA), which is currently being managed medically with methotrexate. The patient is able to actively extend the thumb, index, and middle fingers. When the small and ring fingers are passively extended, the patient is unable to hold them in extension. The proximal forearm muscle bellies have palpable contraction on attempted small and ring finger extension.

## QUESTIONS

1. **What is the differential diagnosis for loss of active finger extension in a patient with rheumatoid arthritis?**
2. **How can physical examination maneuvers help to make the diagnosis in this case?**
3. **What is the correct diagnosis of the patient presented in the current case report?**
4. **What are the surgical options to regain finger extension in this patient?**
5. **What are the implications of this patient being on rheumatoid arthritis medications and how are they best managed in the perioperative period?**

## DISCUSSION

The differential diagnosis of loss of active finger extension in a patient with RA includes attritional tendon rupture, metacarpophalangeal (MP) joint dislocation, subluxation of the extensor tendons into the “valleys” between the metacarpal heads, and denervation of the extensor muscles due to posterior interosseous nerve (PIN) compression at the elbow.^1^ Attritional rupture is caused by constant friction of the tendon as it rubs over bony prominences, direct invasion of the tendon by synovitis, and ischemic necrosis secondary to proliferative synovitis. When rupture occurs, it is typically painless. The tendons most commonly affected by attritional rupture are the extensors on the ulnar side of the hand, which may progress radially in what is known as the Vaughn-Jackson syndrome.

Dislocation of the MP joint results from chronic synovitis and ligamentous laxity. Arthritis of the MP joints is the most common hand complaint associated with RA, but loss of finger extension is an uncommon presentation. More common symptoms are pain, swelling, and deformity, with associated MP joint subluxation and ulnar drift. Frank dislocation of the MP joint is uncommon. The diagnosis is made radiographically.

Ligamentous laxity of the MP joint together with synovitis of the extensor tendons can lead to ulnar subluxation of the extensor tendon into the “valleys” between the metacarpal heads. In this subluxed position, the extensor tendons lose their mechanical advantage and become unable to extend the MP joint. This can be differentiated from extensor tendon rupture by passively extending the MP joint, and asking the patient to hold the fingers in extension, also known as Bouvier's test. If there is a structurally intact tendon, extension of the MP joint will reduce the subluxed tendon into its anatomic position, and the patient will be able to passively hold the fingers in extension. Furthermore, there would be a tenodesis effect when the wrist is passively ranged.

Proximal PIN entrapment is a complication of RA, which occurs as a result of synovitis and bony subluxation at the elbow joint and compression at the arcade of Frohse. Denervation of the long extensor muscles results in inability to extend the digits. Posterior interosseous nerve compression can be differentiated from tendon rupture and subluxation by the fact that the thumb is usually affected by PIN syndrome, whereas the extensor pollicis longus is rarely affected by rupture or subluxation.^2^ In addition, muscle contraction may be palpated in the forearm without concomitant distal tendon excursion and joint motion, further confirming an intact neuromuscular unit but loss of more distal extensor function.

Attritional rupture of the small and ring finger extensor tendons is the correct diagnosis of the patient presented. Primary tendon repair is not usually feasible in attritional extensor tendon rupture. Instead, transfer of unaffected, expendable tendons to replace the function of the ruptured tendon is the surgical strategy of choice. In the case of attritional rupture of the small and ring finger extensors, there are 2 good surgical options. The first is to transfer the extensor indicis proprius tendon to the extensor digitorum tendons of the small and ring fingers. The disadvantage of this option is that the small and ring fingers will not be able to be extended independently. The second option is to transfer the extensor indicis proprius to the small finger extensors and to suture the ring finger extensor to the middle finger extensor tendon in an end-to-side fashion. The disadvantages of this second option are that the ring and middle fingers will not be able to be extended independently, and the previously undamaged middle finger extensor is being manipulated. In addition to performing tendon transfers to address the ruptured tendons, measures must be taken to prevent further attritional rupture from occurring to additional tendons. This typically entails addressing the caput ulnae deformity, and performing a synovectomy of the dorsal compartments. Finally, if bony destruction of the metacarpophalangeal joint progressed, a total arthroplasty should be considered with a silicone implant. Furthermore, medical management of the patient's RA should be optimized.^3^

There are 2 categories of disease-modifying antirheumatic drugs: small molecule drugs, and newer biologic medications, such as TNF (tumor necrosis factor)-inhibitors and Interleukin 1 (IL-1) antagonists. Small molecule drugs, such as methotrexate, which the patient was currently using, have been shown to be safe in the perioperative period. There is no identifiable benefit to holding them for surgery, and doing so is thought to increase all joint pain and thereby impair rehabilitation postoperatively.^4^ In contrast, TNF-α inhibitors have been shown to increase the risk of postoperative infections and should be held for 1 month prior to surgery.^5^

## Figures and Tables

**Figure:**
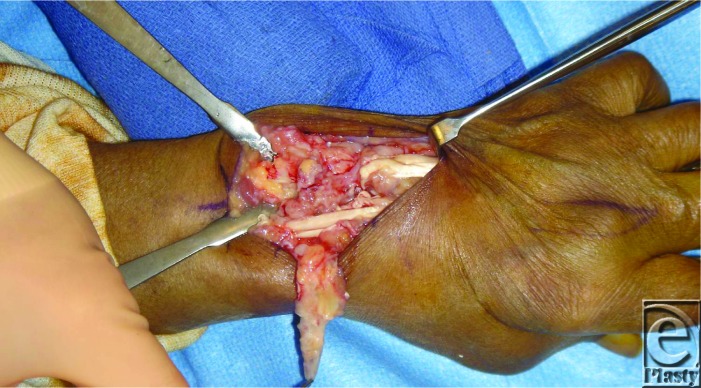


**Figure:**